# Supplementary material for: Peptide signaling without feedback in signal production operates as a true quorum sensing communication system in Bacillus subtilis
Source: Commun Biol. 2021 Jan 8;4:58. doi: 10.1038/s42003-020-01553-5 (PMC7794433; doi:10.1038/s42003-020-01553-5)
Supplement: Supplementary file 2 — Supplementary Information [file 42003_2020_1553_MOESM2_ESM.pdf]

Supplementary Information

Peptide signaling without feedback in signal production operates as a true quorum sensing communication system in *Bacillus subtilis*

Iztok Dogsa[1*], Mihael Spacapan[1], Anna Dragoš[1,2], Tjaša Danevčič[1], Žiga Pandur[1], Ines Mandic-Mulec[1*]

[1] Chair of Microbiology, Department of Food Science and Technology, Biotechnical Faculty, University of Ljubljana, Večna pot 111,1000 Ljubljana, Slovenia EU

[2] Department of Biotechnology and Biomedicine, Section for Microbial and Chemical Ecology, Bacterial Interactions and Evolution, Technical University of Denmark, Søltofts Plads Building: 221, 164, 2800 Kgs. Lyngby, Denmark EU

*Correspondence to: ines.mandicmulec@bf.uni-lj.si, +386-1-3203409 and iztok.dogsa@bf.uni-lj.si, +386-1-3203414

Supplementary Methods

ComX concentration measurements. To measure the concentration of *B. subtilis* signal molecule (SM), i.e. ComX, 12 mL of growing culture was collected from the fermenter at selected time intervals. To remove the cells, the spent medium was centrifuged (5 min, 8000 g) and then filter sterilized. We used spent medium from *B. subtilis* PS-216 (Δ*comP*) as the advantage of this strain is that although its spent medium contains ComX, it does not contain the products that are signal receptor (ComP) dependent and is therefore comparable to the control medium obtained in the parallel experiment, where ComX deficient strain PS-216 (Δ*comQ*) was grown under the same conditions. The dilution series of ComX in spent medium was obtained by mixing spent medium containing ComX with the control medium (Δ*comQ*). For construction of calibration curve the ComX was heterologously expressed in *E. coli* ED367, purified as described in (ref. 1) and diluted in control medium (Δ*comQ*). The quantification of purified ComX was performed as described in section Quantification and isolation of purified ComX.

Final composition of the medium for inoculation by the biosensor *B. subtilis* BD2876 (Δ*comQ*, *srfA-lacZ*) was 50 % (v/v) fresh CM and 50 % (v/v) spent medium that was either dilution of *B. subtilis* PS-216 (Δ*comP*) spent medium in PS-216 (Δ*comQ*) spent medium (5 dilutions, sample curve), or dilution of purified ComX in PS-216 (Δ*comQ*) spent medium (7 dilutions, calibration curve). 200 µL of media for calibration and sample curve were transferred on 96-well microtiter plate, one column (8 replicates) for each ComX concentration. The inoculated plate (2 % inoculum) was placed on a microtiter plate shaker (1 mm orbit, 1000 rpm) that was positioned in the humidified chamber to eliminate sample evaporation. To improve the homogeneity of the conditions (airflow, temperature) on the microtiter plate the microtiter plate lids were elevated by 2 mm relative the surface of microtiter plate. Following incubation at 28 °C for 16 h, we performed β-galactosidase assay. The sufficient response of the biosensor can be obtained also after 4 h of incubation, but it was more practical to obtain the spent media in the morning and early afternoon, prepare the biosensor microtiter plates in the late afternoon, incubate them overnight (16 h) and perform the β-galactosidase assay next morning.

Quantification and isolation of purified ComX. ComX (168 pherotype) was HPLC purified from the *E. coli* ED367 as described previously[1,2]. The obtained ComX concentration was determined from the elution peak on chromatogram by taking into account the estimated theoretical extinction coefficient. For ComX peptide portion we calculated extinction coefficient as described previously[3]. For the value of extinction coefficient of ComX farnesyl portion that chemically corresponds to one half of the squalene, one half of the value for squalene extinction coefficient[4,5] was assumed. The final ComX extinction coefficient used in our calculations was 46300 a.u. mol / cm at $\lambda = 214$ nm. Additionally, we performed MS analysis of HPLC elution fraction containing purified ComX. The elution fraction was deposited on MALDI plate in 0.7 mg / ml HCCA in 85 % ACN, 15 % H2O, 0.1 % TFA and 1 mM $NH_4H_2PO_4$. The analysis was performed on a MALDI-TOF / TOF UltraFlextreme III mass

spectrometer (Bruker, Germany). FlexControl 3.3 software (Bruker, Germany) was used to control the instrument, FlexAnalysis 3.3 to process data and to calibrate spectra (Bruker, Germany). We confirmed the predominant presence of ComX (> 90 %) in the HPLC elution fraction.

To prevent non-specific binding of the ComX filter sterilized BSA was given to the *B. subtilis* spent medium[6] and *E. coli* purified ComX to the final concentration of 50 µg/mL. The purified ComX was typically stored as 200 nM stock solution in SS buffer and kept at 4 °C before use. The stability of stock solutions of purified ComX was periodically checked by HPLC. The obtained ComX concentrations, i.e. the area of biologically active peaks, in aging stock solutions were normalized on initial ComX concentrations of the same stock solutions. The half-life time of ComX was about 50 days (Fig. S6a), which indicates strong chemical stability.

Optimization of growth medium for heterologous expression of ComX in *E. coli* ED367. First modification of original ComX M9 production medium[2] was addition of $FeSO_4$ to the final concentration of 10 µM. This improved the ComX yield significantly (P = 0.004, t-value = 4.6, two-sided), for about 2-fold compared to the original medium (Fig. S6b). To further increase the yield of ComX in *E. coli* ED367, we adopted the previous protocol[7] for very high yields of recombinant proteins in *E. coli* and gained altogether significant 5-fold increase (P < 0.0001, t-value= 9.6, two-sided) compared to the original medium. The overnight LB *E. coli* ED367 culture, supplemented with 100 µg/mL of Amp, grown in 37 °C (200 rpm), was transferred to fresh TB medium (1 % inoculum) supplemented by 20 mM $MgCl_2$ and 100 µg/mL of Amp. After about 2 h and 45 min of incubation at 37 °C (200 rpm) when culture reached OD650 of approximately 10 a.u., the spent growth medium was removed by culture centrifugation (5000 g, 5 min, room temperature). The pellet was re-suspended in the equal volume of fresh optimized M9 growth medium (1x M9 minimal salts (M6030, Sigma-Aldrich, USA), 5 mM $MgSO_4$, 200 µM $CaCl_2$, 1 % glucose, 0.0025 x BME Vitamins 100x solution (B6891, Sigma Aldrich, USA), 10 mg/L leucine, 10 mg/L phenylalanine, 10 mg/L serine, 100 mg/L L-glutamic acid (monosodium salt), 40 mg/L histidine, 40 mg/L methionine, 0.00025 x trace elements 1000 x solution that contained 0.5 x 0.1 M $FeCl_3$ in 0.12 M HCl, 20 mM $CaCl_2$, 10 mM $MnCl_2$, 10 mM $ZnSO_4$, 2 mM $CoCl_2$, 2 mM $CuCl_2$, 2 mM $NiCl_2$, 2 mM Na_2MoO_4, 2 mM Na_2SeO_3, 2 mM H_3BO_3), supplemented by 100 µg/mL of Amp and incubated at 37 °C (200 rpm). When OD650 increased for about 1.0 a.u. the ComX expression was induced by adding IPTG to the final concentration of 0.4 mM and incubation was continued for 4 h. Then the cells were removed by centrifugation (8000 g, 10 min) followed by filtration (pore size 0.2 µm). Sterile supernatant was stored at 4 °C for further use.

β-galactosidase assay. We adapted the original β-galactosidase assay[8] for direct microtiter plate use. Briefly, after determining the culture density (OD650), 25 µL of 5.6 % (v/v) β-mercaptoethanol in Z-buffer and 10 µL of toluene were added to each well and the plates were incubated on ice for 30 min. To improve the sensitivity of the assay, the plates were then warmed to 30 °C, 25 µL ortho-nitrophenyl-β-galactoside (ONPG) substrate (8 mg/mL) was added and the absorbance (420 nm) in 1 min intervals was read using a Multiscan Spectrum

Microplate Reader (Thermo Scientific) operating at 30 °C. From OD650 and kinetics of ONPG degradation the β-galactosidase activity was calculated. The average of the β-galactosidase activity was calculated from at least 8 wells on a microtiter plate. Outliers in OD650 or kinetics were excluded based on the absolute value of modified Z-score > 3.5 as recommended[9]. Prior to Z-score calculation the data was logarithmized in order to obtain normalized distribution.

Exoprotease concentration measurements. *B. subtilis* PS-216 wt was grown in liquid CM[10] or MSgg[11] growth medium at 37 °C and 200 rpm for 24 h. After incubation cells were harvested by centrifugation (10 min at 10000 g and room temperature) and spent medium was filter-sterilized (pores size 0.2 µm). To determine protease concentration, the sterile spent media were diluted in 10 mM sodium acetate buffer with 5 mM calcium acetate (Ca, Na – acetate buffer, pH 7.5) for 10 – 100 times[12]. The undiluted and diluted samples (100 µL) were then dispensed into the wells on the casein gelatin agar plates[12,13]. The plates were incubated for 20 h at 37 °C, then photographed and the diameters of the proteolytic zones were calculated using image analysis by Fiji-ImageJ[14]. To estimate the exoprotease concentration in spent media, the proteolytic zone diameters of the samples were compared to the proteolytic zone diameters obtained by different concentrations of subtilisin (Sigma-Aldrich, USA). The calibration curve was made using subtilisin in Ca, Na - acetate buffer in the range from 0.55 µg/mL to 6.25 µg/mL.

Calculation of ATP requirements for the synthesis of 1 signal molecule (SM) of ComX, 168 pherotype. By assuming carbon source is glucose and that for 1 mol of glucose bacterium obtains 26 mol of ATP[15] one arrives to the conclusion that for synthesis of amino acids for oligopeptide (ADPITRQWGD) in ComX 263 molecules of ATP are required. However, for each ComX molecule a 55 amino acid pre-peptide must be synthesized. Although one can assume that remaining 45 amino acids of the pre-peptide that are not part of the final oligopeptide are recycled, the energy required for 54 peptide bonds in the pre-peptide is significant- for each peptide bond 1 ATP is converted to 1 AMP, 1 ATP to 1 ADP and 2 GTP to 2 GDP, which makes together an equivalent of 4 ATP molecules per peptide bond; for all the bonds in 55 amino acid pre-peptide this makes 216 ATP molecules. Further energy is required for synthesis of farnesyl that is attached to oligopeptide. Farnesyl is made from 3 isoprenoid units that are in *B. subtilis* synthetized via Methylerythritol Phosphate Pathway[16]. For two units of isoprenoids 3 ATP molecules are required[17], hence ~ 5 ATP molecules are needed for synthesis of farnesyl. Taken together, for the synthesis of ComX 484 ATP molecules are required, assuming any other energy costs are negligible. Note that even if one assumes that basic precursors (i.e. amino acids) are already available in the medium, the metabolic cost of ComX remains 221 ATP.

Derivation of ComQXPA communication system model

1. **Signal molecule (SM) production**

One can express the concentration of an arbitrary molecule (*x*) being produced in the closed system as a mathematical product of production rate per cell, $P_x(t)$, the number of producing

cells per unit volume (cell density), *N(t)*, and the time cells were producing the molecule, *t*. The molecule of interest can potentially get degraded by the degradation rate *γ(t)*. In the differential form and as a function of time one can express concentration of signal molecule *SM(t)* as:

$$\frac{dSM(t)}{dt} = P_{SM}(t)\, N(t) - \gamma(t) SM(t) \qquad \text{eq S1}$$

Our previous research showed the degradation of SM (ComX) is possible by *B. subtilis* proteases. But this occurred at significant level only after incubating ComX for 24 h in MSgg spent medium obtained after 24 h of incubation with PS-216 wt (when the proteases accumulated in enough high concentration). In our case, however, the spent media were maximal 7 h old and the protease content of spent CM growth medium used in current study shows 100-times lower concentration compared to MSgg medium used in our previous study (Supplementary Fig. 7). Therefore, we assumed that biological degradation of SM (ComX) is negligible under conditions in this study. Under the buffered conditions (as in CM growth medium), the ComX appears to be stable (Supplementary Fig. 6a) at least in the time frame of experiments where we applied our model. Therefore, in our modeling we assumed that degradation rate of SM is negligible ($\gamma(t) \approx 0$) and the eq S1 can be simplified to:

$$\frac{dSM(t)}{dt} = P_{SM}(t)\, N(t) \qquad \text{eq S2}$$

The $P_{SM}(t)$, SM production rate per cell, incorporates all possible factors influencing the signal synthesis rate such as, the gene regulation and enzyme modulation. In our modeling we are not trying to model the exact molecular mechanism, but are rather seeking the relationship among core quorum sensing (QS) parameters i.e. signal molecule concentration, SM and cell density, $N(t)$. A straightforward option to express rate of signal production per cell, $P_{SM}(t)$, is to assume that $P_{SM}(t)$ simply follows the cell growth, more precisely, specific cell growth rate, $\frac{dN(t)}{N(t)dt}$. The logic behind this assumption is that cell protein content has to increase proportionally to cell growth and SM might be one such molecule. Such simple relationship also implies no special regulation of SM production is present i.e. constitutive SM expression. In this case the rate of signal production per cell, $P_{SM}(t)$, can be expressed in terms of specific cell growth rate as:

$$P_{SM}(t) = a\frac{dN(t)}{N(t)dt}, \qquad \text{eq S3}$$

where a is a proportionality constant that will later serve as fitting parameter and ensures equality.

After combining eq S2 with eq S3, assuming *N(0)* ≈ 0 and then integrating one arrives to:

$$SM(t) = aN(t) \qquad \text{eq S4}$$

Linear relationship of *SM(t)* and *N(t)* was also theoretically predicted by Drees *et al.*[18], who assumed that the production of signal molecule (ComX) per cell $P_{SM}(t)$ is constant and therefore not dependent on cell density or incubation time. The assumption makes sense as long as the cells divide at a constant rate, as in the exponential growth phase and no regulation on SM production is present. This scenario is covered by eq S3 by setting specific cell growth rate to constant and by setting cell density, *N(t)* in eq S2 to increase exponentially (by $2^{t/tgen}$, where *tgen* is generation time), which gives after integration the obtained linear relationship of *SM(t)* and *N(t)*.
This simple linear relation (eq S4), however, does not fit the data in Fig. 2b, as the relationship of cell density and SM is non-linearly increasing, suggesting additional factors to specific cell growth rate are influencing $P_{SM}(t)$. A simplest way to introduce the possible additional factor is to say that SM production rate can be faster or slower from specific cell growth rate and that this weight factor is linearly related to the cell density, $N(t)$. This implies control over SM production and also that the cells producing SM can somehow sense the increased cell density. This is different than in the case of SM feedback regulated QS autoinducing systems where cells producing SM sense increase in SM and respond by increasing the SM production. In our case the rate of signal production per cell, $P_{SM}(t)$ that includes the weight factor $N(t)$ for specific cell growth rate is given by:

$$P_{SM}(t) = a\frac{dN(t)}{N(t)dt}\,N(t) = a\frac{dN(t)}{dt}, \qquad \text{eq S5}$$

where $\frac{dN(t)}{dt}$ is the population growth rate, the measure of how quickly population of bacteria expands in time.

After combining eq S2 with eq S5, assuming $N(t0) \approx 0$ and then integrating, one arrives to:

$$SM(t) = \; a\,N(t)^2, \qquad \text{eq S6}$$

which is indeed the relationship we obtained experimentally in Fig. 2b, where our measurements of *B. subtilis* ComX concentrations show the power b = (2.09 ± 0.10). We also reanalyzed published data from Bacon Schneider *et al.*[19] with eq 1 (main text) and found b = (1.4 ± 0.4), supporting the power-law relationship between bacterial density and SM concentration. The slight difference in the b values could be because we directly related measured ComX concentration to bacterial density, while Bacon Schneider *et al.*[19] related the activity of the spent medium containing unknown amount of ComX to the cell density. In addition, we use here undomesticated strain *B. subtilis* PS-216 while Bacon Schneider *et al.*[19] performed their analyses with the domesticated strain JH642.

2. **Response model**

Analogously to the case of signal molecule production (eq S2) one can also consider the production of response molecule, *RM(t)* as:

eq S7

$$\frac{dRM(t)}{dt} = P_{RM}(t)\,N(t),$$

where $N(t)$ is cell density and $P_{RM}(t)$ is a production rate of a response molecule per cell. As in the case of signal molecule production (eq S3) we assumed that the rate of response molecule production depends on specific cell growth rate and some weight factor. In this case we know that for bacterial communication to work, the response has to follow the signal, which means that $P_{RM}(t)$ has to depend on signal molecule concentration, SM. This relationship is displayed in Fig. 4a, where the signal molecule concentration (SM) was the only varying condition and the response to SM was measured and fitted by the model equation, response sensitivity to SM:

$$W(SM) = \frac{\mathrm{W}_{max}\,SM^n}{\mathrm{Km}^n + SM^n}\,, \qquad \text{(eq 2, main text)}$$

where *SM* is the concentration of signal molecule and *Km* is the SM concentration at which half of the maximum response is achieved; *n* (Hill coefficient) describes the cooperativity among transcriptional activators[20,21]. Therefore, we can express production rate of a response molecule production per cell, $P_{RM}(t)$, analogously to eq S5, except that we use *W(SM)* as a weight factor of specific cell growth rate:

$$P_{RM}(t) = k\,\frac{dN(t)}{N(t)dt}\,W(SM) \qquad \text{eq S8}$$

with *k* as proportionality constant and *W(SM)* response sensitivity to the signal molecule, SM. By combining (eq S6) with (eq 2) one can express the response sensitivity to the signal molecule in terms of bacterial density *N*(*t*) as:

$$W(SM) = \frac{\mathrm{Wmax}(aN(\mathrm{t})^2)^n}{\mathrm{Km}^n + (aN(\mathrm{t})^2)^n} \qquad \text{eq S9}$$

Inserting (eq S9) into (eq S8) and then into (eq S7) gives than the differential form for the response molecule concentration in time:

$$dRM(t) = k\,\frac{\mathrm{Wmax}(a\mathrm{N(t)}^2)^n}{\mathrm{Km}^n + (a\mathrm{N(t)}^2)^n}\,dN(t) \qquad \text{eq S10}$$

After integration of the above equation one obtains the following expression:

$$RM1(t) = \frac{k\ \mathrm{Wmax}\ a^{n}\mathrm{Km}^{-n}(\mathrm{N(t)}^{2n+1})\mathrm{Hyperg2F1}\left[1{,}1+\frac{1}{2n}{,}2+\frac{1}{2n}{,}-\left(\frac{a}{\mathrm{Km}}\right)^{n}\mathrm{N(t)}^{2n}\right]}{1+2n} \quad \text{eq S11}$$

Here Hyperg2F1 is a hypergeometric function $_2F_1$ that is defined as:

$$_2F_1(x, y; w; z) = \sum_{j=0}^{\infty}(x)_j\,(y)_j/(w)_j\, z^j/j! \quad \text{eq S12}$$

Eq S11 is however not our final fitting equation for response to SM during bacterial growth in the closed system (Fig. 4b). One can expect some left overs of response molecules (β-galactosidase units) in the cells that accumulated in the overnight culture that served as a direct inoculum. The concentration of this initial response molecules, RM0, contributes to the RM(t). Furthermore, we are experimentally determining β-galactosidase activity that is a measure of β-galactosidase units per cell i.e. we are measuring the concentration of response molecules per cell, $\frac{RM(t)}{N(t)}$. Accounting for initial concentration of response molecules per cell, $\frac{RM0}{N(t)}$ and newly produced ones, $\frac{RM1(t)}{N(t)}$, we can express the total concentration of response molecules per cell as:

$$\frac{RM(t)}{N(t)} = \frac{RM0}{N(t)} + \frac{RM1(t)}{N(t)} \quad \text{(eq 3, main text)}$$

Regarding $\frac{RM(t)}{N(t)}$ as a measured β-galactosidase activity (Fig. 4b), the eq 3 with the reference to eq S11 was our final fitting equation. Note that for the purpose of fitting the N(t) was expressed as the optical density of bacterial culture (OD650). As our fitting procedure by eq 3 required a continuous function of OD650, the measured OD650 was fitted by logistic equation:

$$\mathrm{OD}_{650}(\mathrm{t}) = \mathrm{A2} + \frac{(A1-A2)}{1+\left(\frac{\mathrm{t}}{\mathrm{t0}}\right)^{p}} \quad \text{eq S13}$$

where *t* is time of bacterial growth, *A1* the lower horizontal asymptote and *A2* the upper horizontal asymptote, *t0* is the time denoting midpoint of growth and *p* describes the steepness of the growth curve.

Supplementary Figures and Tables

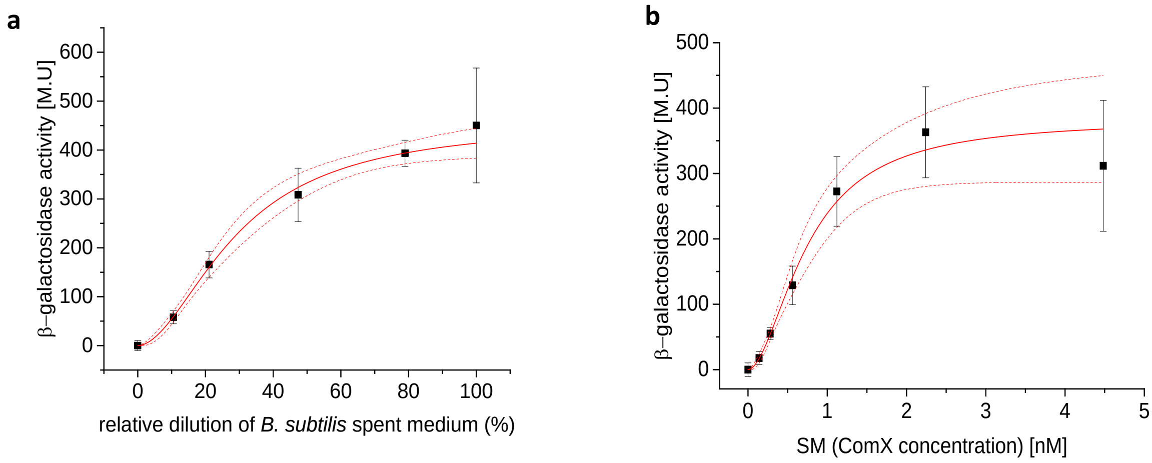

Supplementary Figure 1: ComX concentration determination via biosensor assay. During the growth of *B. subtilis* PS-216 (*ΔcomP*, ComX producer, - does not respond to the ComX) the spent medium containing unknown amount of ComX was collected and diluted in spent medium of *B. subtilis* PS-216 (*ΔcomQ* – does not produce ComX) and tested for the presence of ComX by the biosensor *B. subtilis* BD2876 (*ΔcomQ*- does not produce ComX, responds to ComX via expression of *srfA-lacZ*). **a** An example of the biosensor BD2876 response to the spent medium of *B. subtilis* PS-216 (*ΔcomP*) measured as a β-galactosidase activity is given. **b** For ComX concentration determination the calibration curve was obtained in parallel - the HPLC purified ComX was diluted in spent medium of *B. subtilis* PS-216 (*ΔcomQ*) and then tested by the biosensor BD2876. Each point represents the average of 8 wells on the microtiter plate, error bars represent SD. (red line) best fit by eq 2 with 95 % confidence level (dotted red line). Only the linear parts of the curves were considered for ComX concentration calculations that was done by comparing BD2876 response in (**a**) to (**b**). In the above example, there is 3 nM of ComX in tested *B. subtilis* spent medium.

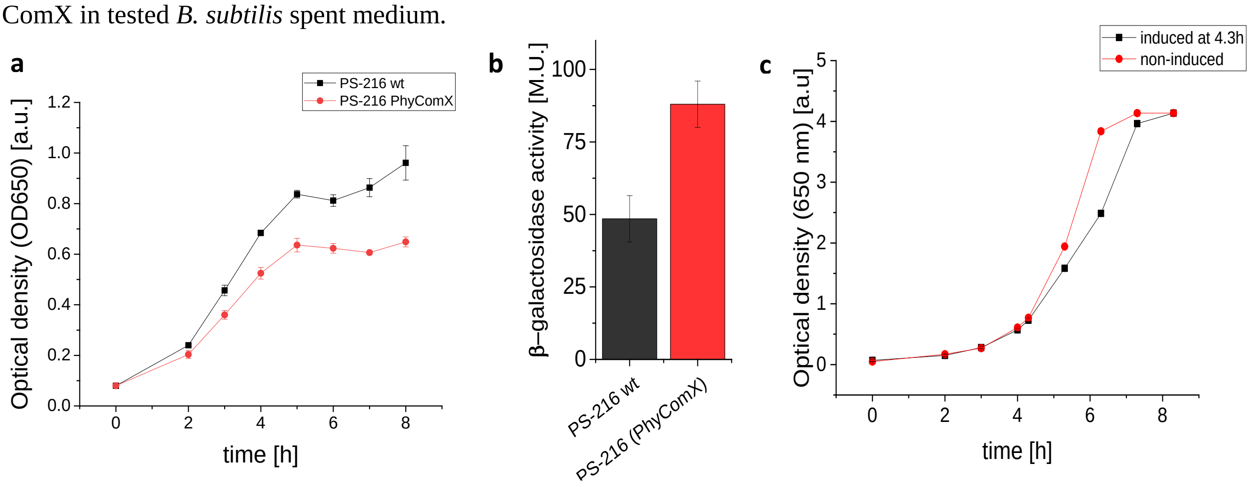

Supplementary Figure 2. The fitness cost of overexpressed ComX production. **a** The growth curves of *B. subtilis* PS-216 wt and PS-216 (*PhyComX*) carrying additional copy of *comX* under control of IPTG-inducible promoter. Both strains were grown in presence of 1 mM IPTG. Data points represent average from n = 3 independent biological replicates, error bars represent standard error. **b** To verify ComX overproduction by PS-216 (*PhyComX*), the activity of spent media from PS-216 wt and PS-216 (*PhyComX*) was tested using biosensor strain BD2876 (Δ*comQ srfA-lacZ).* Data points represent average from n = 3 independent biological replicates, error bars represent SD. **c** The growth curves of *E. coli* ED367 with the induction of recombinant signal molecule production (ComX) after 4.3 h, or without induction. One of the five qualitatively and quantitatively similar experiments is presented.

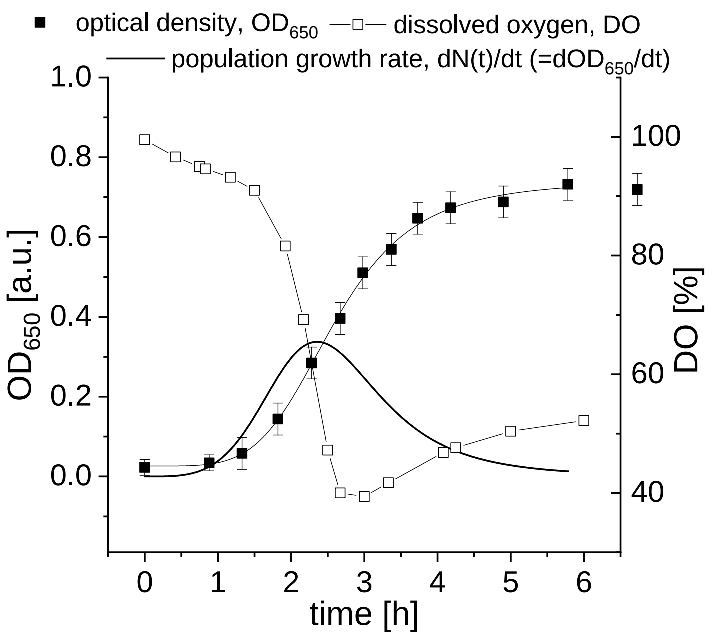

Supplementary Figure 3: The growth curve (black squares) of *B. subtilis* PS-216 wt and the dissolved oxygen (white squares), DO, during growth in the fermenter working in the batch mode. One of the three qualitatively and quantitatively similar experimental replicates is presented. The population growth rate (black line) (eq S5). The error bars represent estimated uncertainty basec on 5 technical replicates of the OD650 measurements.

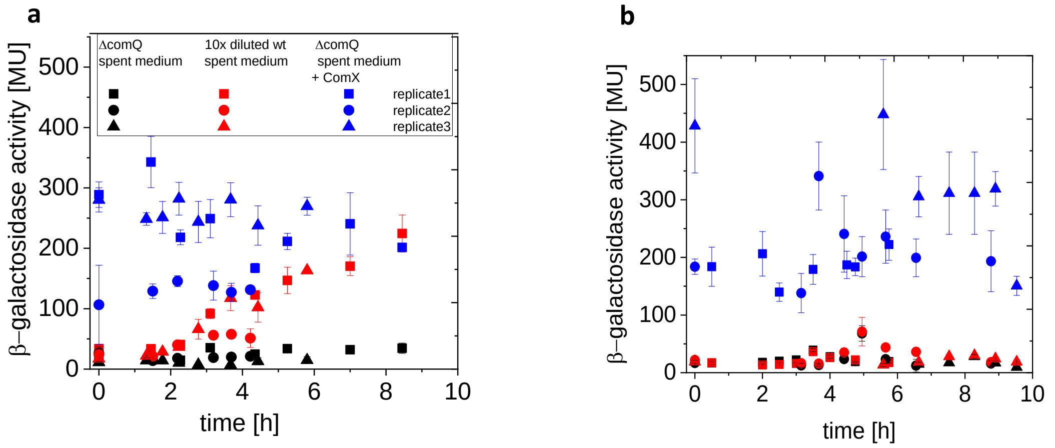

Supplementary Figure 4: β-galactosidase activity of the ComX biosensor BD2876 and the influence of O2 on the presence of SM (ComX). ComX biosensor BD2876 was incubated in the fresh CM medium supplemented with either spent medium of the *ΔcomQ* strain (no ComX, negative control, black symbols) or supplemented with spent medium of the PS-216 (*ΔcomQ)* strain with added isolated ComX (≈1 nM, positive control, blue symbols) or supplemented with the wt strain spent medium 10 times diluted by spent medium of the *ΔcomQ* strain (experimental, red symbols); the shape of the symbol corresponds to n = 3 independent experiments. The spent medium of the PS-216 (*ΔcomQ)* strain was obtained in the parallel fermenter batch system. The strains PS-216 wt and PS-216 (*ΔcomQ)* were grown in the fermenter batch system where oxygen supply was limited (**a**) or supplied to the saturation (**b**). The ComX biosensor BD2876 barely responded to the spent medium with no ComX (PS-216 *ΔcomQ* spent medium) and strongly responded to the same medium when purified ComX was added, indicating that ComX is the major factor controlling *srfA-lacZ* expression in biosensor BD2876. However, the same quantity of ComX added to the spent media collected at different time points does not induce the same level of response by biosensor, therefore for quantification of ComX (Fig 2a,b) the *ΔcomQ* spent medium used for construction of calibration curve was collected for each time point separately (see Material and methods, Biosensor based quantification of ComX concentration).

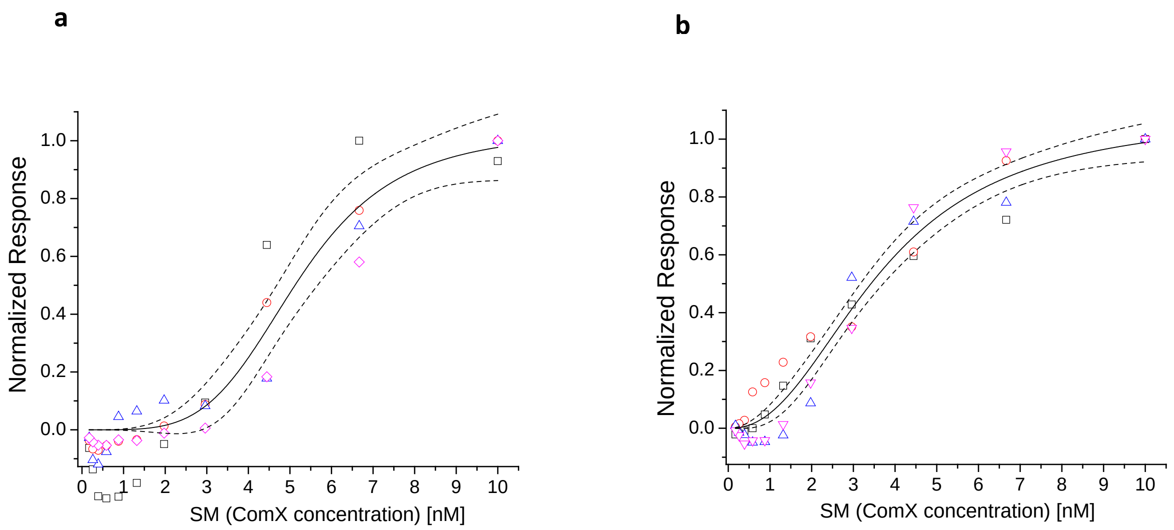

Supplementary Figure 5: Maximum normalized response measured as the *srfA* promoter activity of *B. subtilis* PS-216 (Δ*comQ*, P_{srfAA}-*yfp*) (no signal production) to the exogenously added signal molecule, SM, (ComX). The strain was incubated in the presence of SM for 3 hours (**a**), and 6 hours (**b**). n = 4 independent experiments are shown, where each type of the symbol corresponds to one replicate. Best, concatenated, fits (black line) to the model eq 2 are presented together with 95 % confidence level (black dotted line) for each time point.

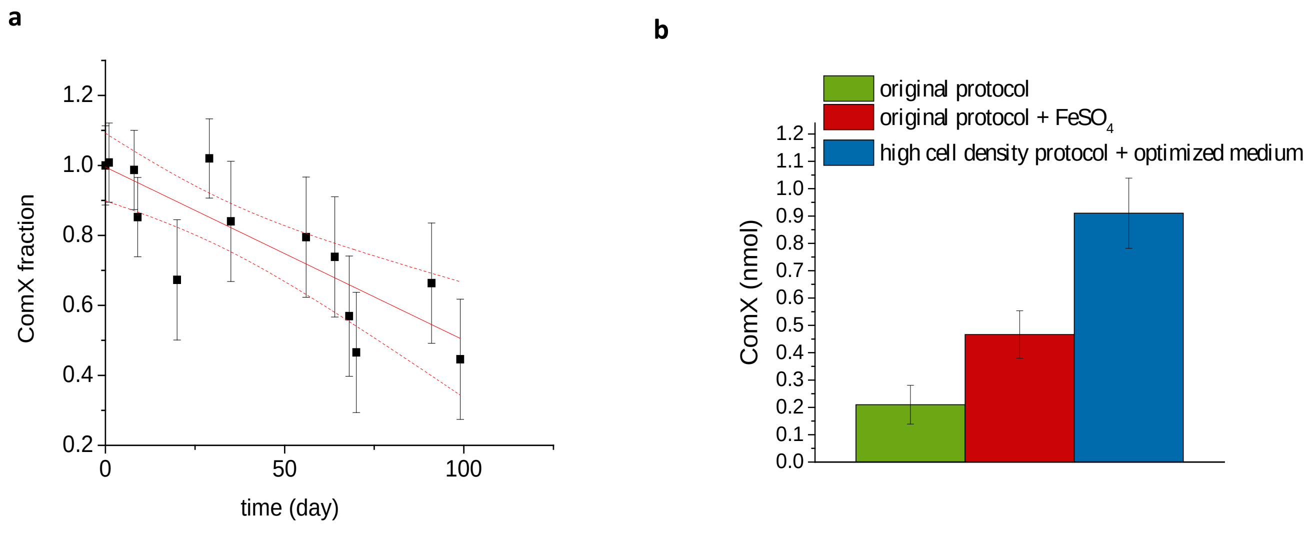

Supplementary Figure 6: The stability and yields of purified ComX. a The aging of purified ComX stored in SS buffer at 4 °C as measured by HPLC. The data was obtained during n = 8 independent experiments; the error bars represent SD estimate of technical replicates. Red line represents linear fit to the data. **b** Yields of ComX per mL of *E. coli* ED367 spent medium obtained by three different protocols. The production medium M9 in original protocol[2] by was supplemented by $FeSO_4$. Additional improvements were achieved by switching to high cell density protocol and further medium optimization (see Optimization of growth medium for heterologous expression of ComX in *E. coli* ED367 for details). Four independent experiments were conducted, error bars represent SD.

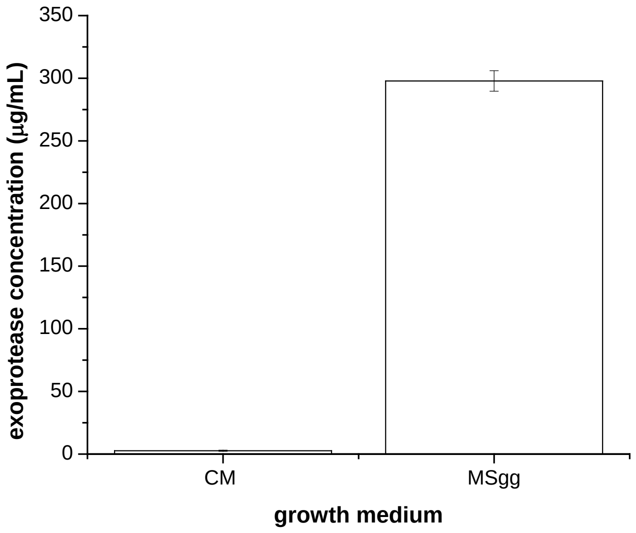

Supplementary Figure 7: Exoprotease concentration in the spent media after growing *Bacillus subtilis* PS-216 wt in liquid growth medium for 24 h at 37 °C. CM medium as used in this study is compared to MSgg medium as used in the study by Spacapan *et al.*[22], where it was shown that the proteases of PS-216 wt can degrade ComX. The protease content of CM growth medium used in this study is drastically reduced compared to MSgg medium. n =3 independent experiments were conducted, error bars represent SD.

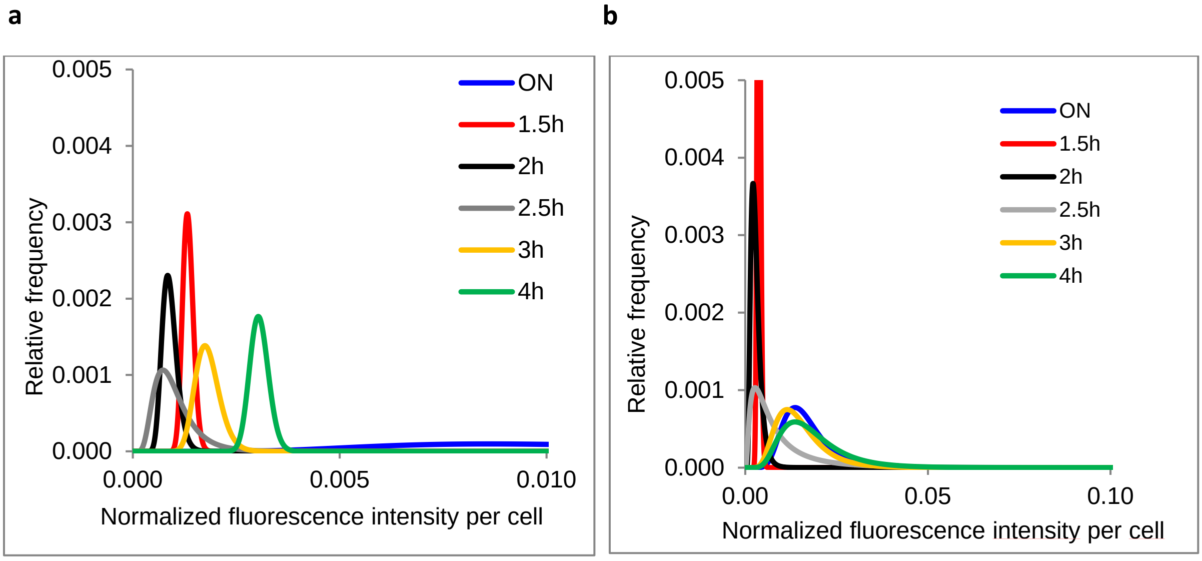

Supplementary Figure 8: The second replicate (for first replicate, see Fig 5g,h) of the distribution of gene expression measured by single cell fluorescence microscopy. Gene expression level was measured as Na-fluoresceinate standard normalized mean fluorescence intensity per cells expressing *comQ-yfp* (**a**) and *srfA-cfp* (**b**). ON is the overnight culture; areas under the curves are the same in all time points.

Supplementary Table 1: Strains used in this study

Name (culture collection ID, strain abbreviation)	genotype	source
Bacillus subtilis PS-216 wt	Undomesticated wild-type isolate	(23)
BM1297 *B. subtilis* PS-216 (*srfA-cfp, comQ-yfp*)	*srfA-cfp (cat) amyE::PcomQ-yfp (sp)*	This work
BM1402 *B. subtilis* PS-216 (*ΔcomP*)	*comP*::cat (*cat*)	(1)
BM1418 *B. subtilis* PS-216 (*ΔcomQXP*)	*comQXP*::*kan* (kan)	(1)
BM1692 *B. subtilis* PS-216 (*srfA-lacZ*)	PS-216 wt (*srfA-lacZ*) (neo)	(24)
BM1455 *B. subtilis* PS-216 (Δ*comQ*, P_{srfAA}-*yfp*)	Δ*comQ amyE::*P_{srfAA}-*yfp (sp)*	(25)
BM1042 *B. subtilis* PS-216 (*PhyComX*)	*srfA-cfp (cat) thrC::Pspac-hy-comX (mls)*	This work
BM1400 *B. subtilis* PS-216 (*ΔcomQ*)	*comQ::kan*	(22)
BM1154 *B. subtilis* BD 2876 *(*Δ*comQ, srfA-lacZ*)	his leu met *srfA-lacZ* (tet) *comQ::kan*	(26)
EM1048 *E. coli* ED367	F- ompT gal dcm lon hsdSB(rB- mB-) λ(DE3 [lacI lacUV5-T7 gene1 ind1 sam7 nin5]) pET22)	(2)

Name (culture collection ID, strain abbreviation)	role (SM = signal molecule, ComX)
Bacillus subtilis PS-216 wt	SM producer and responder to SM
BM1297 *B. subtilis* PS-216 (*srfA-cfp, comQ-yfp*)	SM producer and responder to SM, *cfp* and *yfp* reporters
BM1402 *B. subtilis* PS-216 (*ΔcomP*)	SM producer, cannot respond to SM
BM1418 *B. subtilis* PS-216 (*ΔcomQXP*)	cannot produce SM, cannot respond to SM
BM1692 *B. subtilis* PS-216 (*srfA-lacZ*)	SM producer and responder to SM, *lacZ* response reporter
BM1455 *B. subtilis* PS-216 (Δ*comQ, PsrfA-yfp*)	cannot produce SM, can respond to SM, *yfp* response reporter
BM1042 *B. subtilis* PS-216 (*PhyComX*)	IPTG inducible hyperproducer of SM, can respond to SM
BM1400 *B. subtilis* PS-216 (*ΔcomQ*)	cannot produce SM, can respond to SM
BM1154 *B. subtilis* BD 2876 *(*Δ*comQ, srfA-lacZ*)	biosensor, cannot produce SM, can respond to SM, *lacZ* reporter
EM1048 *E. coli* ED367	recombinant producer of SM for SM isolation

Supplementary Table 2: The values of fitting parameters of the eq 2 to the experimental data of maximum normalized response (Fig. 4a, Supplementary Fig. 3). Standard error and the quality of fit, reduced χ2 is shown; *n* is the level of the cooperativity of the SM, *Km* the concertation of SM at the half-maximum response and *Wmax* the saturation level of normalized response.

	**3h**	**4h**	**6h**
Wmax	1.03±0.10	1.10±0.05	1.09±0.07
Km [nM]	5.2±0.4	3.5±0.2	3.7±0.3
n	4.4±1.0	2.2±0.2	2.3±0.3
reduced χ^2	0.01	0.003	0.005

Supplementary Table 3: The average values of fitting parameters values of fit of 3 growth curves of PS-216 *(srfA-lacZ)* by eq S13 (see Fig. 4b). The R^2 of all fits was ≥ 0.99 and reduced $\chi^2 < 3 \times 10^{-4}$. Relatively small standard deviation indicates the growth curves were very similar.

parameter	Value	Standard deviation
A1	0.029	0.004
A2	0.75	0.02
t0	2.61	0.05
p	4.8	0.5

Supplementary Table 4: The average values of fitting parameters values and statistics of fits of 5 experimental sets by eq 3 (see Fig. 4b), which represents the ComQXPA communication system model. T-test was two-sided. The R^2 of all fits was ≥ 0.95. Relatively small standard deviation indicates the response curves were very similar.

parameter	value	Standard deviation	t-statistic	P-value
k	760	120	>15	$<1\times10^{-7}$
RM0	5.5	1.5	>9	$<7\times10^{-6}$

Supplementary Table 5: Correlation of the *srfA-cfp* with *comQ-yfp* expression determined in the same PS-216 cells during incubation time. ON means overnight culture. The correlation is expressed as an average ± SD of Pearson correlation coefficient obtained from 3 independent experiments via co-localization analysis of images taken by the single cell fluorescence microscopy. The Control represents the auto-fluorescence correlation between the CFP and YFP channel of the PS-216 wt strain (without fluorescence markers); Experimental represents the correlation of the fluorescence intensities between the CFP and YFP channel of the PS-216 strain (with fluorescence markers). Note that when measuring the fluorescence in Experimental samples one obtains the auto-fluorescence and marker fluorescence together. The fluorescence intensity of the two markers could not be measured independently. Therefore, the extraction of the true distributions of their fluorescence intensity was performed by mathematical deconvolution. The obtained fluorescence intensity log-normal distributions were randomly added to the Control to simulate the fluorescence intensity of the Experimental sample where no correlation among CFP and YFP is present (Simulated Experimental zero correlation prediction). At 3-4 h, when *comQ* and *srfA* are well expressed (Fig. 5) the Experimental has the same correlation coefficient than the Control and significantly larger correlation ($P = 0.01$; t-value = 4.5, two-sided) than Simulated Experimental zero correlation prediction, indicating the presence of positive correlation of about 0.5 for the correlation of *srfA-cfp* expression with *comQ-yfp* expression in the same cell. The correlation of the Control is in all analyzed time points roughly constant, on the other hand correlation in Experimental drops with time and becomes indistinguishable from the Simulated Experimental zero correlation prediction indicting that the correlation of *srfA-cfp* with *comQ-yfp* expression is lost in the overnight culture.

	**Correlation of:**	**3-4h**	**5-6h**	**ON**
Control	autofluorochromes	0.53 ± 0.05	0.59 ± 0.03	0.64 ± 0.20
Simulated Experimental zero correlation prediction	autofluorochromes+ Cfp and Yfp uncorrelated	0.17 ± 0.11	0.27 ± 0.07	0.11 ± 0.11
Experimental	autofluorochromes+ Cfp correlated Yfp	0.51 ± 0.07	0.47 ± 0.16	0.19 ± 0.22

Supplementary References

1 Oslizlo A, Stefanic P, Dogsa I, and Mandic-Mulec I. Private link between signal and response in Bacillus subtilis quorum sensing. PNAS 2014; 111:1586-1591.

2 Ansaldi M, Marolt D, Stebe T, Mandic-Mulec I, Dubnau D. Specific activation of the Bacillus quorum-sensing systems by isoprenylated pheromone variants. Mol Microbiol 2002; 44:1561–1573.

3 Kuipers, B. J. & Gruppen, H. Prediction of molar extinction coefficients of proteins and peptides using UV absorption of the constituent amino acids at 214 nm to enable quantitative reverse phase high-performance liquid chromatography-mass spectrometry analysis. J. Agric. Food. Chem. 55, 5445-5451. (2007).

4 Jarstfer, M. B., Zhang D. L. & Poulter, C. D. Recombinant squalene synthase. Synthesis of non-head-to-tail isoprenoids in the absence of NADPH. JACS 124: 8834-8845 (2002).

5 Pittler, S. J., Fliesler, S.J., Fisher, P. L., Keller, P. K. & Rapp, L. M. In vivo requirement of protein prenylation for maintenance of retinal cytoarchitecture and photoreceptor structure. J. Cell. Biol. 130, 431-439 (1995).

6 Magnuson, R., Solomon, J. & Grossman, A. D. Biochemical and genetic characterization of a competence pheromone from B. subtilis. Cell. 77, 207–216 (1994).

7 Sivashanmugam, A., Murray, V., Cui, C., Zhang, Y., Wang, J. & Li, Q. Practical protocols for production of very high yields of recombinant proteins using Escherichia coli. Protein. Sci. 18, 936-948 (2009).

8 Miller, J. H. Experiments in Molecular Genetics. (Cold Spring Harbor Laboratory Press: Cold Spring Harbor, USA) (1972).

9 Iglewicz, B. & Hoaglin, D. Volume 16: How to Detect and Handle Outliers. (ASQC Quality Press: Milwaukee, USA) (1993).

10 Albano, M., Hahn, J. & Dubnau, D. Expression of competence genes in Bacillus subtilis. J. Bacteriol. 169, 3110–3117 (1978).

11 Branda, S. S., González-Pastor, J. E., Ben-Yehuda, S., Losick, R. & Kolter, R. Fruiting body formation by Bacillus subtilis. Proc. Natl Acad. Sci. 98, 11621–11626 (2001).

12 Griffith, K. L & Grossman, A. D. A degenerate tri-partite DNA binding site required for activation of ComA-dependent quorum response gene expression in Bacillus subtilis. J. Mol. Biol. 381, 261–275 (2008).

13 Montville, T. J. Dual-substrate plate diffusion assay for proteases. Appl. Environ. Microbiol. 45, 200 – 204 (1983).

14 Schindelin, J., et al. Fiji: an open-source platform for biological-image analysis. *Nat. Methods.* **9**, 676-682 (2012).

15 Kaleta, C., Schäuble, S., Rinas, U. & Schuster, S. Metabolic costs of amino acid and protein production in Escherichia coli. Biotechnol. J. 8, 1105–1114 (2013).

16 Wagner, W. P., Helmig, D. & Fall, R. Isoprene Biosynthesis in Bacillus subtilis via the Methylerythritol Phosphate Pathway. J. Nat. Prod. 63, 37-40 (2000).

17 Banerjee, A. & Sharkey, T. D. Methylerythritol 4-phosphate (MEP) pathway metabolic regulation. Nat. Prod. Rep., 31, 1043-1055 (2014).

18 Drees, B., Reiger, M., Jung. K. & Bischofs, I. B. A modular view of the diversity of cell-density-encoding schemes in bacterial quorum-sensing systems. Biophys. J., 107, 266-277 (2014).

19 Bacon Schneider, K., Palmer, T. M. & Grossman, A. D. Characterization of comQ and comX, two genes required for production of ComX pheromone in Bacillus subtilis. J. Bacteriol. 184, 410–419 (2002).

20 Alon, U. An Introduction to Systems Biology: Design Principles of Biological Circuits (Chapman & Hall / CRC: Boca Raton, USA) (2007).

21 Chu, D., Zabet, N. R., Mitavskiy, B. Models of transcription factor binding: Sensitivity of activation functions to model assumptions. J. Theor. Biol. 257, 419–429 (2009).

22 Spacapan M, Danevčič T, Mandic-Mulec I. ComX-Induced Exoproteases Degrade ComX in Bacillus subtilis PS-216. Front Microbiol 2018; 9:105. doi: 10.3389/fmicb.2018.00105

23 Stefanic P, Mandic-Mulec I. Social interactions and distribution of Bacillus subtilis pherotypes at microscale. J Bacteriol 2009; 191: 1756–1764.

24 Danevčič T, Borić Vezjak M, Tabor M, Zorec M and Stopar D. Prodigiosin Induces Autolysins in Actively Grown Bacillus subtilis Cells. Front Microbiol 2016; 7: 27. doi: 10.3389/fmicb.2016.00027

25 Spacapan M, Danevčič T, Stefanic P, Porter M., Stanley-Wall N.R., Mandic-Mulec I. The ComX quorum sensing peptide of Bacillus subtilis affects biofilm formation negatively and sporulation positively. Microorgansims 2020; 8, 1131-1151.

26 Tortosa P, Logsdon L, Kraigher B, Itoh Y, Mandic-Mulec I, Dubnau D. Specificity and Genetic Polymorphism of the Bacillus Competence Quorum-Sensing System. J Bacteriol 2001; 183: 451–460.
