# Supplementary material for: Peptide signaling without feedback in signal production operates as a true quorum sensing communication system in Bacillus subtilis
Source: Commun Biol. 2021 Jan 8;4:58. doi: 10.1038/s42003-020-01553-5 (PMC7794433; doi:10.1038/s42003-020-01553-5)
Supplement: Supplementary file 3 — Description of Additional Supplementary Files [file 42003_2020_1553_MOESM3_ESM.pdf]

Description of Additional Supplementary Files

File Name: Supplementary Data 1
Description: The source data underlying figures. Each sheet corresponds to one figure
