# Supplementary material for: Peptide signaling without feedback in signal production operates as a true quorum sensing communication system in Bacillus subtilis
Source: Commun Biol. 2021 Jan 8;4:58. doi: 10.1038/s42003-020-01553-5 (PMC7794433; doi:10.1038/s42003-020-01553-5)
Supplement: Supplementary file 5 — Reporting Summary [file 42003_2020_1553_MOESM5_ESM.pdf]

nature research

Corresponding author(s): Iztok Dogsa

Last updated by author(s): Nov 1, 2020

Reporting Summary

Nature Research wishes to improve the reproducibility of the work that we publish. This form provides structure for consistency and transparency in reporting. For further information on Nature Research policies, see our Editorial Policies and the Editorial Policy Checklist.

Statistics

For all statistical analyses, confirm that the following items are present in the figure legend, table legend, main text, or Methods section.

n/a	Confirmed	
☐	☒	The exact sample size (*n*) for each experimental group/condition, given as a discrete number and unit of measurement
☐	☒	A statement on whether measurements were taken from distinct samples or whether the same sample was measured repeatedly
☐	☒	The statistical test(s) used AND whether they are one- or two-sided<br>*Only common tests should be described solely by name; describe more complex techniques in the Methods section.*
☐	☒	A description of all covariates tested
☐	☒	A description of any assumptions or corrections, such as tests of normality and adjustment for multiple comparisons
☐	☒	A full description of the statistical parameters including central tendency (e.g. means) or other basic estimates (e.g. regression coefficient) AND variation (e.g. standard deviation) or associated estimates of uncertainty (e.g. confidence intervals)
☐	☒	For null hypothesis testing, the test statistic (e.g. *F*, *t*, *r*) with confidence intervals, effect sizes, degrees of freedom and *P* value noted<br>*Give P values as exact values whenever suitable.*
☒	☐	For Bayesian analysis, information on the choice of priors and Markov chain Monte Carlo settings
☒	☐	For hierarchical and complex designs, identification of the appropriate level for tests and full reporting of outcomes
☐	☒	Estimates of effect sizes (e.g. Cohen's *d*, Pearson's *r*), indicating how they were calculated

Our web collection on statistics for biologists contains articles on many of the points above.

Software and code

Policy information about availability of computer code

Data collection	ZEN 2.3 (blue edition) (Carl Zeiss GMBH, Germany), SkanIt Software 2.2 (Thermo Scientific, Inc., USA), Eurochrom 3.05 P4 (Knauer GMBH, Germany) , custom
Data analysis	OriginPro (OriginLab, USA), Wolfram Mathematica 11.0 (Wolfram Research, Inc., USA), Fiji ImageJ (Wayne Resband, NIH, USA), custom code was deposited to Github, https://doi.org/10.5281/zenodo.4205585 , https://doi.org/10.5281/zenodo.4206230

For manuscripts utilizing custom algorithms or software that are central to the research but not yet described in published literature, software must be made available to editors and reviewers. We strongly encourage code deposition in a community repository (e.g. GitHub). See the Nature Research guidelines for submitting code & software for further information.

Data

Policy information about availability of data

All manuscripts must include a data availability statement. This statement should provide the following information, where applicable:

- Accession codes, unique identifiers, or web links for publicly available datasets
- A list of figures that have associated raw data
- A description of any restrictions on data availability

The data that support the findings of this study are available from the corresponding author upon reasonable request.

Field-specific reporting

Please select the one below that is the best fit for your research. If you are not sure, read the appropriate sections before making your selection.

- [x] Life sciences
- [ ] Behavioural & social sciences
- [ ] Ecological, evolutionary & environmental sciences

For a reference copy of the document with all sections, see nature.com/documents/nr-reporting-summary-flat.pdf

Life sciences study design

All studies must disclose on these points even when the disclosure is negative.

Sample size	In general, in microbiology the variability of the obtained results is not the consequence of low number of tested subjects bacteria as these are always numerous. It is rather the imprecise control of the conditions under which bacteria are grown or tested. To amend for this we used mini-fermenter system with precise computer controlled stirring, temperature regulation and oxygen supply and experiments were performed in at least three biological replicates performed on different days. If experiments were performed in microtiter plates, at least 8 wells were used as replicates within a biological replicate. In experiments with larger volumes at least two Erlenmeyer flasks were used within an experiment. On top of that, we always performed all our experiments in at least three independent (different days) experiments.
Data exclusions	In microplate assays we performed statistical analysis by using modified Z-score to exclude the outliers. If the data was strongly at odds with other replicates, we only excluded it, if it was clear that this was due to the error of the experimenter. For example, in microscopy, if an image taken was clearly out of focus, it was excluded from the evaluation. We always took care that wrongly performed experiments were double replaced by correctly performed experiments.
Replication	Reproducibility of the experimental findings was verified by making several technical and at least three independent (different days) experiments. In addition, important findings were investigated by two methods or persons and checked for consistency.
Randomization	This is not applicable to bacterial cultures as these are always very numerous.
Blinding	The full blinding of the investigators is very difficult to achieve in the laboratory type experiments usually performed in microbiology as the experimenter sets the conditions to which bacteria are exposed. The objectivity was however strengthened by using quantitative analysis using digital equipment and software. Also, different parts of our research were performed by different people who did not know what is the favorable outcome for the central story of the research. Furthermore, some parts of the research were replicated by different methods by researchers who did not know the results of the previous outcome. The subjects of the investigations (bacteria) were, of course, blind.

Reporting for specific materials, systems and methods

We require information from authors about some types of materials, experimental systems and methods used in many studies. Here, indicate whether each material, system or method listed is relevant to your study. If you are not sure if a list item applies to your research, read the appropriate section before selecting a response.

Materials & experimental systems

n/a	Involved in the study	
☒	☐	Antibodies
☒	☐	Eukaryotic cell lines
☒	☐	Palaeontology and archaeology
☒	☐	Animals and other organisms
☒	☐	Human research participants
☒	☐	Clinical data
☒	☐	Dual use research of concern

Methods

n/a	Involved in the study	
☒	☐	ChIP-seq
☒	☐	Flow cytometry
☒	☐	MRI-based neuroimaging
